# *Fasciola hepatica* soluble antigens (*Fh*Ag) induce ovine PMN innate immune reactions and NET formation in vitro and in vivo

**DOI:** 10.1186/s13567-023-01236-z

**Published:** 2023-11-12

**Authors:** Tamara Muñoz-Caro, Marcela Gómez-Ceruti, Liliana M. R. Silva, Daniel Gutiérrez-Expósito, Henrik Wagner, Anja Taubert, Carlos Hermosilla

**Affiliations:** 1 Escuela de Medicina Veterinaria, Facultad de Medicina Veterinaria y Recursos Naturales, Universidad Santo Tomás, Talca, Chile; 2 Centro de Investigación de Ovinos Para El Secano OVISNOVA, Facultad de Medicina Veterinaria y Recursos Naturales, Universidad Santo Tomás, Talca, Chile; 3 Centro de Investigação Interdisciplinar Egas Moniz (CiiEM), Instituto Universitário Egas Moniz, Caparica, Portugal; 4 MED-Mediterranean Institute for Agriculture, Environment and Development & CHANGE-Global Change and Sustainability Institute, Universidade de Évora, Evora, Portugal; 5 Departamento de Sanidad Animal, Instituto de Ganadería de Montaña (CSIC-ULE), Facultad de Veterinaria, Universidad de León, Campus de Vegazana s/n, 24071 León, Spain; 6 Veterinary Clinic for Reproduction and Neonatology, Justus Liebig University Giessen, Giessen, Germany; 7 Institute of Parasitology, Faculty of Veterinary Medicine, Justus Liebig University Giessen, Giessen, Germany

**Keywords:** *Fasciola hepatica*, neutrophil extracellular traps, NETosis, ROS, innate immunity, sheep, livestock

## Abstract

*Fasciola hepatica* causes liver fluke disease, a worldwide neglected and re-emerging zoonotic disease, leading to hepatitis in humans and livestock. In the pathogenesis, flukes actively migrate through liver parenchyma provoking tissue damage. Here, parasites must confront leukocytes of the innate immune system in vivo. Polymorphonuclear neutrophils (PMN) are the most abundant granulocytes and first ones arriving at infection sites. PMN may display neutrophil extracellular traps (NETs), consisting of nuclear DNA, decorated with histones, enzymes, and antimicrobial peptides. We investigated for the first time whether *F. hepatica* soluble antigens (*Fh*Ag) can also trigger NETosis and innate immune reactions in exposed ovine PMN. Thus, isolated PMN were co-cultured with *Fh*Ag and NET formation was visualized by immunofluorescence and scanning electron microscopy analyses resulting in various phenotypes with spread NETs being the most detected in vitro. In line, NETs quantification via Picogreen®-fluorometric measurements revealed induction of anchored- and cell free NETs phenotypes. Live cell 3D-holotomographic microscopy revealed degranulation of stimulated PMN at 30 min exposure to *Fh*Ag. Functional PMN chemotaxis assays showed a significant increase of PMN migration (*p* = 0.010) and intracellular ROS production significantly increased throughout time (*p* = 0.028). Contrary, metabolic activities profiles of *Fh*Ag-exposed PMN did not significantly increase. Finally, in vivo histopathological analysis on *F. hepatica*-parasitized liver tissue sections of sheep showed multifocal infiltration of inflammatory cells within liver parenchyma, and further fluorescence microscopy analyses confirmed NETs formation in vivo. Overall, we hypothesized that NET-formation is a relevant host defence mechanism that might have a role in the pathogenesis of fasciolosis in vivo.

**Supplementary Information:**

The online version contains supplementary material available at 10.1186/s13567-023-01236-z.

## Introduction

*Fasciola hepatica* is the causative agent of fasciolosis, a worldwide neglected and re-emerging zoonotic disease [1]. This trematode species affects a wide range of wild and domestic vertebrate species, predominantly sheep and cattle, in regions with temperate climates [2]. Human fasciolosis, is also a major plant/food-borne zoonosis worldwide [3] with up to 17 million infected people worldwide, thereby representing a public health relevance in several regions of Asia, South America, Europe and Africa [1, 4].

In livestock animals, this gastropod-borne trematode disease causes important economic losses associated with reduced livestock milk/meat production, morbidity, and mortality due to hepatitis, cholangitis and cholecystitis [5], with an estimated annual cost of €2.5 billion globally in the livestock production industry [6, 7]. In the pathogenesis of fasciolosis migrating juvenile flukes as well as adults play a pivotal role. As such, juvenile flukes must actively migrate through the liver parenchyma in order to reach bile ducts where they mature into adult stage parasites [1] becoming blood feeders [8]. Acute fasciolosis is especially problematic in sheep when large numbers of migrating juvenile flukes invade the liver inducing parenchyma destruction, pro-inflammatory reactions, and bleeding. Clinical signs of ovine fasciolosis include anaemia, ascites, and abdominal pain, which are also associated with sub-acute disease characterised by severe hepatic haemorrhage leading in some cases to sudden death [9, 10]. Meanwhile, chronic fasciolosis, the most common clinical presentation, might lead to emaciation, especially in more susceptible animals and in ewes in the advanced stages of gestation [10].

The initial infection control against *F. hepatica* is provided by host innate immune leukocytes such as polymorphonuclear neutrophils (PMN), monocytes, eosinophils, and macrophages. These leukocytes become activated after initial contact with migrating *F. hepatica* parasites through pathogen recognition receptors (PRRs) binding to parasite-specific molecules in order to hamper parasite development in vivo [11, 12]. Despite the pivotal role of leukocytes during fasciolosis in vivo, very little is known on early interactions between PMN and *F. hepatica* [11], even though PMN are the first granulocytes to be recruited to infection sites [13]. Consistently, PMN are the most abundant leukocytes circulating in the blood/lymph system and playing a crucial role in development of innate immune and adaptive immune responses [14], via phagocytosis, secretion of pro-inflammatory cytokines/chemokines, production of reactive oxygen species (ROS) and degranulation of antimicrobial peptides and proteins [15–18]. Additionally, a more recently described effector mechanism of PMN, and of other leukocytes, is the formation of extracellular traps (ETs), an innate defence mechanism to fight invading pathogens [19, 20] including protozoan and metazoan parasites [21]. NETs are mainly composed of nuclear DNA decorated with granule proteins, such as histones (H1, H2A/H2B, H3, H4), neutrophil elastase (NE), myeloperoxidase (MPO), lactoferrin, gelatinase, pentraxin, and cathelicidin among others [22–24]. During NETosis, thin fiber-like extracellular structures can be extruded, bearing capacity not only for entrapping invading pathogens but also for killing them [20, 25–27].

Large-sized and highly motile nematode species such as *Strongyloides stercoralis* [28], *Haemonchus contortus* [29], *Nippostrongylus brasiliensis* [30], *Brugia malayi* [31], *Dirofilaria immitis* [32], *Ostertagia ostertagi* [33] and *Angiostrongylus vasorum* [34] seem likewise capable to trigger strong NETosis reactions not only in vitro but also in vivo [13, 21]. Conversely, only three reports exist so far in literature on trematode-induced NETosis, i.e. *Schistosoma japonicum* [35], *Fasciola gigantica* [36] and *F. hepatica* in the bovine system [37]. Therefore, the aim of the present study was to elucidate for first time whether *F. hepatica*-soluble antigen (*Fh*Ag) is able to induce early immune interactions in ovine PMN and NET formation via in vitro and in vivo approaches.

## Materials and methods

### Isolation of ovine PMN

Healthy adult Suffolk sheep (*n* = 6) were bled by puncture of the jugular vein and peripheral blood was collected in BD Vacutainer® heparin tubes (Franklin Lakes, USA). Approximately 20 mL of heparinized blood were diluted in 20 mL sterile PBS with 0.02% EDTA (Sigma-Aldrich, St. Louis, USA), layered on top of 12 mL Biocoll® separating solution (density = 1.077 g/L; Sigma-Aldrich) and centrifuged (800 × *g*, 45 min). After removal of plasma and peripheral blood mononuclear cells (PBMC), the cell pellet was suspended in sterile Hanks’ Balanced Salt Solution 1X (HBSS; Gibco, Thermo Fisher Scientific, Waltham, MA, USA) for subsequent lysis of ovine erythrocytes by using lysis buffer solution (0.3 mM NaH_2_PO_4_, 0.4 mM KH_2_PO_4_, all Merck, Burlington, Massachusetts, USA) for 1 min at room temperature (RT). Then, a hypertonic buffer [0.3 mM NaH_2_PO_4_, 0.4 mM KH2PO4, 0.136 mM NaCl (all Merck)] and HBSS were added to the solution and centrifuged at 600 × *g* for 10 min. This process was repeated twice. Finally, the pellet was washed in sterile HBSS solution at 600×*g* for 10 min two times. PMN were re-suspended in sterile HBSS medium (Gibco), counted in a Neubauer haemocytometer chamber, and left on ice to rest (30 min) before use.

### Soluble *Fasciola hepatica* antigen (*Fh*Ag) preparation

For preparation of soluble *Fh*Ag the protocol of Peixoto et al. [37] was followed: four alive *F. hepatica* adults collected from the liver of naturally infected cattle were collected at a local butchery and immediately transported at 4 °C to the Laboratory of Veterinary Parasitology of Universidad Santo Tomás, Talca, Chile, and immediately frozen in liquid nitrogen. Thereafter frozen parasites were grounded in a previously UV-sterilized and cooled mortar (−80 °C for 1 h) for soluble *Fh*Ag preparation. Thus, 300 μL sterile phosphate-buffered saline (PBS; 1X; Sigma-Aldrich) were added into a mortar and collected parasites were added, grounded and the suspension was then sonicated in an ice bath with a digital ultrasonic bath Biobase UC-20A® sonicator for five cycles of 15 s. The sonicated material was then centrifuged at 10 000 × *g* for 20 min at 4 °C. The protein concentration of supernatant was measured using the method of BCA® protein assay (Thermo Fisher Scientific) according to manufacturer instructions and final *Fh*Ag solutions were stored at −20 °C until further use.

### Immunofluorescence microscopy analyses for visualization of *Fh*Ag-induced NETosis in vitro

Analysis of NET formation induced by *Fh*Ag was also assessed in vitro. Here, ovine PMN (*n* = 3) diluted in sterile serum-free RPMI 1640 cell culture medium (Sigma-Aldrich) without phenol red were seeded on previously poly-l-lysine (0.01%; Sigma-Aldrich)-treated glass coverslips in a plastic 24-well plate (Greiner, Kremsmünster, Austria). Ovine PMN (2 × 10^5^ PMN) were stimulated with 100 µg/mL of *Fh*Ag according to Peixoto et al. [37], plain medium as negative control and PAF (platelet-activating factor; 100 nm) as positive control at 37 °C, 5% CO_2_ for 120 min incubation. Then, cells were fixed with 4% (w/v) paraformaldehyde (Merck) for 20 min, at RT, washed thrice with PBS, and stored at 4 °C until further use. After fixation, cells were analysed for detection of pan-histones [anti-histones (H1, H2A/H2B, H3, H4) antibody (MAB3422 Sigma-Aldrich; 1:200)] or anti-NE antibody (AB68672 Abcam, Cambridge, UK; 1:200) and incubated at 4 °C overnight. Then, samples were washed twice with sterile PBS and incubated in respective second conjugated antibody solution [anti-mouse Alexa Fluor 594 A-11005; 1:500 and anti-rabbit Alexa Fluor 488 A-11008, 1:500 dissolved in buffer (PBS 1X, 3% BSA, 0.3 Triton X-100)] for 1 h at RT in the dark. Then, specimens were washed thrice with sterile PBS and mounted in Fluoromount-G® with DAPI (Thermo Fischer Scientific) for 24 h at RT in the dark. Visualization of NET structures based on co-localized extracellular DNA staining and histone- and NE-derived signals, was achieved by using an inverted Olympus IX81® epifluorescence microscope equipped with a XM10® digital camera (Olympus, Hamburg, Germany). Finally, based on same experimental settings, visual quantification of PMN undergoing NETosis was achieved by taking three different power vision pictures from each experimental condition (*n* = 3) on 20× magnification by using an inverted IX81® fluorescence microscope equipped with an XM 10® digital camera (both Olympus). Microscopic identification and quantification of NETotic cells was performed following protocol of de Buhr et al. [38] for quantification of the PMN number and the induced ET forming cells with occurrence of distinct extracellular off-shoot of the cells positive for histone-DNA complexes. Results were expressed as percentage of NETotic cells with respect to the total cells positive to DAPI staining per condition.

### Scanning electron microscopy (SEM) analysis

Freshly isolated ovine PMN (*n* = 3) were stimulated either with soluble *Fh*Ag (100 μg/mL) or plain medium as negative control, for 2 h according to Peixoto et al. [37] on coverslips pre-coated with 0.01% poly-l-lysine (Sigma-Aldrich) in an incubator at 37 °C and 5% CO_2_ atmosphere. After incubation, cells were fixed in 2.5% glutaraldehyde (Merck), then post-fixed in 1% osmium tetroxide (Merck), washed in distilled water and dehydrated, dried by critical point CO_2_-treatment and sputtered with gold particles [39]. Samples were visualized via a Philips XL30® scanning electron microscope at the Institute of Anatomy and Cell Biology of the Faculty of Human Medicine, Justus Liebig University Giessen, Giessen, Germany.

### Extracellular DNA-based quantification of cell free- and anchored NETs induced by *Fh*Ag

Cell free- and anchored-NETs determination was performed according to Tanaka et al. [40] demonstrating that extruded NETs can be divided into two distinct forms: (i) NETs being released away and without contact to ruptured PMN, named cell-free NETs, and (ii) released NETs but still anchored to ruptured PMN, named anchored NETs. Briefly, ovine PMN (2 × 10^5^; *n* = 3) were resuspended in sterile serum-free RPMI 1640 media without phenol red (Sigma-Aldrich) and co-cultured for 120 min with soluble *Fh*Ag (10 or 100 μg/mL) at 37 °C and 5% CO_2_ atmosphere in 96-well plastic flat-bottom plates (Greiner). After incubation, the culture supernatant of each well (100 µL) was collected, and thereafter transferred to another well for quantification of cell free NETs by PicoGreen®-based fluorometric measurements. After removal of supernatants, fresh medium was added to each well and PicoGreen®-based fluorometric measurements was also used to detect anchored NETs attached to the bottom of culture wells. For both sampling methods, a 1:200 dilution of PicoGreen® (Invitrogen) in 10 mM Tris base buffered with 1 mM EDTA was added to each well (50 μL), and then extracellular DNA was detected and quantified by PicoGreen®-derived fluorescence intensities via spectrofluorometric analysis by using an automated multiplate monochrome reader (Varioskan Flash®; Thermo Scientific) at 484 nm excitation/520 nm emission. As controls, PMN alone, PMN treated with saponin (1% w/v; Sigma‐Aldrich) for assessment of total DNA, PMN treated with DNase (Thermo Fisher Scientific) to dissolve NETs, and PMN exposed to zymosan 1 mg/mL (Sigma-Aldrich) as positive control were used.

### Live cell imaging of ovine PMN exposed to *Fh*Ag

Ovine PMN (1 × 10^6^; *n* = 3) were centrifuged at 300 × *g* for 10 min at RT, and cells were thereafter suspended in 2 mL of pre-warmed imaging medium [sterile RPMI 1640 cell culture medium without phenol red (Sigma-Aldrich) containing Hoechst (1:1000) to label DNA and SYTOX® Green (1:2000)]. For 3D holotomography, ovine labelled ovine PMN were seeded into 35 mm low-rimmed tissue culture μ-dishes (Ibidi®, Gewerbehof, Germany) and allowed to settle for 10–15 min. Before stimulation with soluble *Fh*Ag (100 μg/mL), images were acquired every 1 min using a 3D Cell Explorer- fluo microscope (Nanolive®, Tolochenaz, Switzerland) equipped with 60 × magnification (λ = 520 nm, sample exposure 0.2 mW/mm^2^) and a depth of field of 30 μm. To maintain cells under controlled conditions [i. e. 37 °C, 5% CO_2_, an Ibidi® top-stage chamber (Ibidi, Gewerbehof, Germany)] was used. Immediately after stimulation, images were acquired every min for 16–17 h by using the same settings. At the end of the live cell imaging experiments, obtained images were analysed using Steve® software v.2.6 (Nanolive®, Tolochenaz, Switzerland) to obtain refractive index (RI)-based z-stacks. Further, 3D rendering and digital staining were performed based on RI values and thereafter illustrated. Additionally, each channel was exported separately using Steve® software v.2.6 (Nanolive®, Tolochenaz, Switzerland) and managed with Image J Fiji® v154e (NIH, Bethesda, MD, USA).

### In vitro ovine PMN chemotaxis assay

In vitro ovine PMN chemotaxis assay was performed following protocols of Conejeros et al. [41] with minor changes. Cell culture insert strips (BRANDplates®) with 3 μm diameter pores were previously hydrated for 5 min with sterile cell culture RPMI 1640 medium without phenol red (Sigma-Aldrich) at 37 °C, 5% CO_2_. In a final volume of 500 μL, 100 nM PAF (Merck) as positive control, plain cell culture RPMI 1640 medium (negative control) or *Fh*Ag (100 μg/mL) in cell culture RPMI medium were added to plastic 24-well cell culture plates (BRANDplates®). Ovine PMN (5 × 10^5^ per condition; *n* = 3) were suspended in 250 μL of cell culture RPMI 1640 medium and carefully layered into the hydrated inserts. The plates were incubated for 120 min (37 °C, 5% CO_2_). After removal of inserts, the number of PMN which migrated to the plate bottom was determined by using an inverted Olympus IX81® epifluorescence microscope equipped with an XM10® digital camera (Olympus).

### Intracellular reactive oxygen species (ROS) production in *Fh*Ag-stimulated ovine PMN

Intracellular ROS production of ovine PMN was assessed by oxidation of 2′,7′-dichlorofluorescein diacetate (DCFH-DA, Sigma-Aldrich) to fluorescent DCF according to Conejeros et al. [41, 42]. In brief, freshly isolated PMN (*n* = 4) were resuspended in sterile 1X HBSS containing Ca^2+^ and incubated with soluble *Fh*Ag (10 or 100 μg/mL) (4 × 10^5^ cells/well; 37 °C, 30 min, in duplicates). Afterwards, DCFH-DA (10 μg/mL) was added to each duplicate. For positive PMN control stimulation, zymosan was used (1 mg/mL; Sigma-Aldrich). The relative fluorescence units (RFU) were detected every 15 min, for a period of 90 min applying 485 nm excitation and 530 nm emission wavelengths (Varioskan® Flash, Thermo Scientific).

### Oxygen consumption rate (OCR), extracellular acidification rate (ECAR) and proton efflux rate (PER) in *Fh*Ag-stimulated ovine PMN

To study ovine PMN mitochondrial activation in terms of OCR, ECAR and PER after soluble *Fh*Ag stimulation, the protocol of Peixoto et al. [37] was applied by using a commercial Seahorse XF® analyser (Agilent, Santa Clara, USA). Briefly, after centrifugation of 1 × 10^6^ isolated ovine PMN (*n* = 3; 500 × *g* for 10 min, at RT), cells were resuspended in 0.5 mL of XF® RPMI 1640 assay medium (Agilent) supplemented with 2 mM of l-glutamine, 1 mM pyruvate and 10 mM glucose (all Sigma-Aldrich). In total, 2 × 10^5^ cells were seeded in 6- of the 8-well Seahorse XF® analyser plates (Agilent) pre-coated for 30 min with 0.001% poly-l-lysine (Sigma-Aldrich). Moreover, on 2 blank wells (without cells), the same volume of XF® RPMI 1640 assay medium (Agilent) (50 μL) was added. For a total volume of 180 μL per well, 130 μL XF® RPMI 1640 assay medium (Agilent) were added carefully and incubated (37 °C, without CO_2_, 45 min). Soluble *Fh*Ag (100 μg/mL) was suspended in XF® RPMI 1640 assay medium (Agilent) (total volume 20 μL) and placed in injection portal B (*n* = 3). For negative controls (*n* = 3), 20 μL plain XF® RPMI 1640 assay medium (Agilent) were added to the same injection portal. For the real time metabolic assay, five basal measurements were included, then either soluble *Fh*Ag or medium were injected, followed by 25 measurements (total run of 180 min). Background subtraction, determination of OCR, ECAR, PER as well as the area under the curve (AUC) of obtained registries were performed by using Wave® software (Desktop Version, Agilent) and GraphPad Prism® version 9.4.1.

### Histopathological analysis of *Fasciola hepatica* infected ovine liver

Three liver samples from naturally *F. hepatica*-infected Suffolk sheep (*n* = 3) were obtained from local slaughterhouse from Santa Cruz city in Chile as well as from a home slaughter within endemic *F. hepatica* sheep farms in Maule Region 35° 25′ 36″ S 71° 40′ 18″ O. Then, four tissue sections were taken from each liver in the dimension of 1 cm^3^ from areas showing gross lesions with emphasizing on the area of bile ducts. Liver sections were withdrawn for immediate fixation in 10% neutral buffered formalin, embedded in paraffin and cut at 2–4 μm for H&E and immunofluorescence staining. H&E staining was routinely processed by using 0.1% hematoxylin (Roth) and 1% eosin (Roth). Further, the H&E slides were examined microscopically on an Olympus CX23 (Olympus) light microscope. Liver samples from healthy sheep were used as negative control.

### Visualization of in vivo NETs induction in *Fasciola hepatica*-parasitized ovine liver

Paraffin blocks used for histopathological analysis were cut at 3 μm tissue sections, mounted and dried on Superfrost Plus® slides (Thermo Scientific). Then, immunodetection of NETs in paraffin-embedded tissue protocol was followed as previously described by Brinkmann et al. [43]. Briefly, after standard dewaxing and rehydration process, sections were incubated in Target Retrieval Solution® pH6 10 mM Citrate 10 × (Dako S236984-3) for 90 min at 50 °C in a water bath. After antigen retrieval, sections were left to cool at RT, rinsed with deionized water three times, TBS pH7.4 one time, and permeabilized for 5 min with 0.5% Triton X100 in TBS (Thermo Fisher Scientific) at RT, followed by three rinsing steps with TBS (Thermo Fisher Scientific). Then sections were surrounded with a PAP®-pen, permeabilized for 10 min (0.1% Triton X-100) and blocked with blocking buffer (BlockAid, Thermofisher Scientific) for 60 min to prevent non-specific binding. Primary antibodies used were anti-histones (H1, H2A/H2B, H3, H4) antibody (MAB3422 Chemico Int) and rabbit anti-neutrophil elastase (NE; Bioss). Both at 1:100 concentration, diluted in blocking buffer and incubated overnight at 4 °C in a humidified chamber. The secondary staining was performed for 1 h in the dark at RT using anti-mouse Alexa Fluor 594 A-11005; 1:500 and anti-rabbit Alexa Fluor 488 A-11008, 1:500 dissolved in blocking buffer. Counterstaining of DNA was performed by using anti-fade mounting medium with DAPI (ProLong® Gold Antifade Reagent, Thermofisher Scientific). The stained samples were examined microscopically on a Leica confocal inverted microscope Stellaris 5® (Leica). Isotype control antibodies were used in separate preparations. Liver samples from healthy sheep were used as negative control. Pictures were edited with ImageJ® software.

### Statistical analyses

Statistical analyses were performed using GraphPad Prism® version 9.5.1, GraphPad® Software, San Diego, California, USA. For comparison of two groups, unpaired two-tailed Mann–Whitney tests were applied. For chemotaxis analysis ratio-paired two-tailed t test was used. For comparing three or more different groups, no pairing non-parametric Kruskal–Wallis test was performed with Dunn’s multiple comparison test. Statistical significance was defined at *p* < 0.05 value.

## Results

### *Fh*Ag induce NETs in exposed ovine PMN in vitro

**Figure 1:**
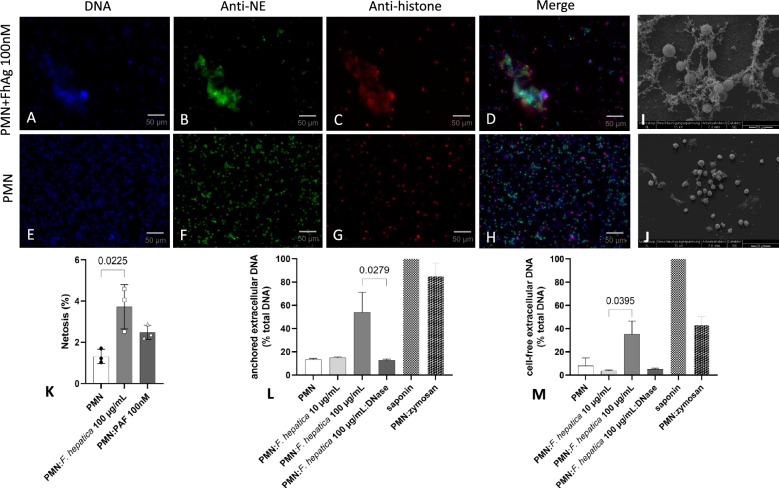
***Fasciola hepatica antigen (FhAg)-induced neutrophil extracellular traps (NETs) formation, analysed via fluorescence microscopy, SEM and PicoGreen®-derived fluorescence intensities.*** Fluorescence microscopy analysis demonstrates co-localization of DNA (**A**, **E**; blue), neutrophil elastase (NE) (**B**, **F**; green) and pan-histones (H1, H2A/H2B, H3, H4) (**C**, **G**; red) of ovine PMN confronted to *Fh*Ag 100 μg/mL. Depiction of the overlay of the three channels (**D**, **H**). SEM analysis confirmed ovine NET formation observing fine spread NETs (*spr*NETs) after exposure to *Fh*Ag 100 μg/mL (**I**). As expected, PMN negative control remained mostly inactivated (**J**). Immunofluorescence analysis shows proportion of ovine PMN undergoing NETosis observing differences of NET formation on PMN exposed to *Fh*Ag 100 μg/mL compared to control (*p* = 0.022) after 120 min of incubation (**K**). PicoGreen®-derived fluorescence intensities analysis shows quantification of anchored NETs observing a significant increase compared with DNase control (*p* = 0.027) at 120 min of incubation (**L**) and an increase on release of cell free NETs induced by *Fh*Ag 100 μg/mL at 120 min incubation when compared to *Fh*Ag 10 μg/mL (*p* = 0.039) (**M**). As controls, PMN alone, PMN treated with saponin (1% w/v) for assessment of total DNA, PMN treated with DNase to dissolve NETs, and PMN exposed to zymosan 1 mg/mL as positive control were here used. For statistical analyses ANOVA with Kruskal–Wallis test was used.

Classical components of NETs were investigated via immunofluorescence microscopy analyses. Co-cultured ovine PMN with soluble *Fh*Ag validated the traditional hallmarks of mammalian-derived NETs proving the presence of extracellular chromatin (Figure 1A, blue), NE (Figure 1B, green) and pan-histone [H1, H2A/H2B, H3, H4] (Figure 1C, red). Thus, the co-localization of extracellular DNA with histones and NE confirmed the presence of typical NETs proteins associated with the chromatin released from ruptured PMN (Figure 1D, merge). Consequently, SEM analysis confirmed *Fh*Ag-induced NET formation on ovine PMN under same concentration of 100 μg/mL used for immunofluorescence analysis (Figure 1I) observing same fine spread NETs (*spr*NETs) after exposure to *Fh*Ag. As expected, PMN negative control remained mostly inactivated (Figure 1J).

Moreover, the percentage of ovine PMN undergoing NETosis was also analysed under microscopic identification showing differences on NET formation (*p* = 0.02; Figure 1K) on PMN exposed to *Fh*Ag compared to control (PMN in plain medium). Nonetheless, the induction of ovine NETosis displayed by this analysis was rather weak reaching a value of 3.7% after 120 min incubation. Ovine PMN exposed to PAF did not promote the release of NETs significantly when compared to PMN control demonstrating that this compound might not be a suitable NET inducer for the ovine system.

### *Fh*Ag triggers formation of anchored- and cell free NETs

Besides microscopic analyses, NETosis quantification of anchored- and cell free-NETs was performed by PicoGreen®-derived fluorescence intensities analysis observing the release of both, cell free- and anchored NETs on PMN exposed to *Fh*Ag in a dose dependent manner (Additional file 1). However, a stronger increase of extracellular DNA release was observed for anchored NETs (Figure 1L) compared to cell free NETs (Figure 1M) in *Fh*Ag 100 μg/mL exposed ovine PMN as shows normalized data to the total DNA release from cells (saponin control) in each case. For anchored NETs, significant differences were observed of extracellular DNA derived from *Fh*Ag 100 μg/mL-exposed ovine PMN at 120 min incubation (*p* = 0.03) compared with DNase control. In contrast, a small proportion of cell free NETs were observed released from *Fh*Ag-exposed PMN when compared to controls. Here, only significant differences were observed between different *Fh*Ag concentrations exposed to PMN (*p* = 0.04). Interestingly, under these experimental settings, zymosan proved to be a suitable positive control for NET induction in ovine PMN.

### Live cell 3D-holotomography illustrated early changes on ovine PMN exposed to *Fh*Ag

Additionally, we used live cell three-dimensional holotomographic microscopy (3D Cell Explorer®, Nanolive, Lausanne, Switzerland) to analyse the changes of ovine PMN induced by *Fh*Ag throughout the dynamic NETotic process. As early as 30 min after parasitic antigen exposure, degranulation of exposed ovine PMN was observed. From this time point, ovine PMN granules were shown disperse in the cytoplasm but from 4 h onwards higher RI signals were observed closer to the nucleus (red rectangle). At 12 h some cells were disintegrated while others remained with a compacted nucleus (Additional file 2).

### *Fh*Ag induced ovine PMN chemotaxis in vitro

**Figure 2:**
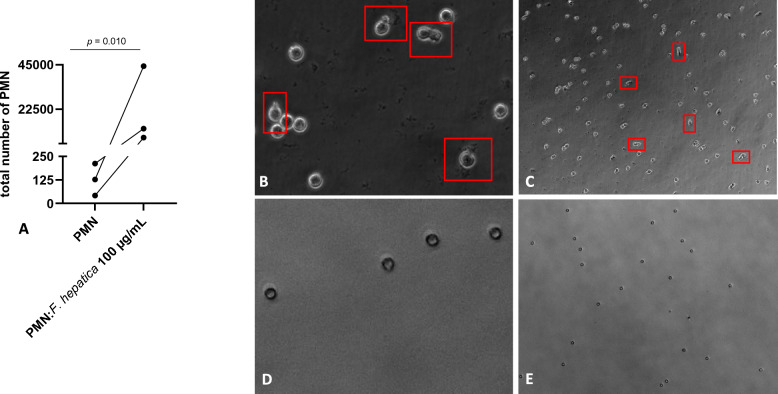
**Chemotaxis of ovine PMN induced by FhAg 100 μg/mL.** **A** Ovine PMN (*n* = 3) were placed in a transwell system and incubated for 120 min to allow PMN migration through 3 μm diameter pores to the bottom of the plate. Plain medium was used as negative control. The graphic represents the number of PMN that migrated to the bottom of the plate. Statistical significance was defined as *p* < 0.05 in ratio paired t-test (*p* = 0.010). **B**, **C** *Fh*Ag induced-PMN chemotaxis displaying PMN with elongated and irregular shapes compatible with activated cells. **D**, **E** negative control showing PMN with rounded cell morphology compatible to non-activated cells.

Once we confirmed early innate immune events of *Fh*Ag exposed to ovine PMN by 3D microscopy, we determined the chemotactic response of PMN to *Fh*Ag stimulation. By using a transwell migration assay (TMA) with *Fh*Ag 100 μg/mL placed at the bottom of the chamber. After TMA incubation, the number of PMN that migrated to the bottom of the chamber were counted. As outcome, we observed a statistically significant increase in the migration of PMN induced by *Fh*Ag (*p* = 0.010; Figure 2A). Plain medium was used as negative control and PAF 100 nM served as positive control. Here, PAF did not significantly induced ovine PMN chemotaxis reaching similar value levels to negative control (plain medium; data not shown). In addition, power vision pictures from migrated PMN into the *Fh*Ag well bottom displayed the presence of activated PMN with elongated and irregular shapes (Figures 2B and C, red boxes) along with PMN showing typical rounded cell morphology compatible to non-activated cells as shown by negative control (Figures 2D and E). Thereby, by this TMA assay, we demonstrate the ability of *Fh*Ag to induce ovine PMN chemotaxis in vitro.

### *Fh*Ag-stimulated ovine PMN increased intracellular ROS production

**Figure 3:**
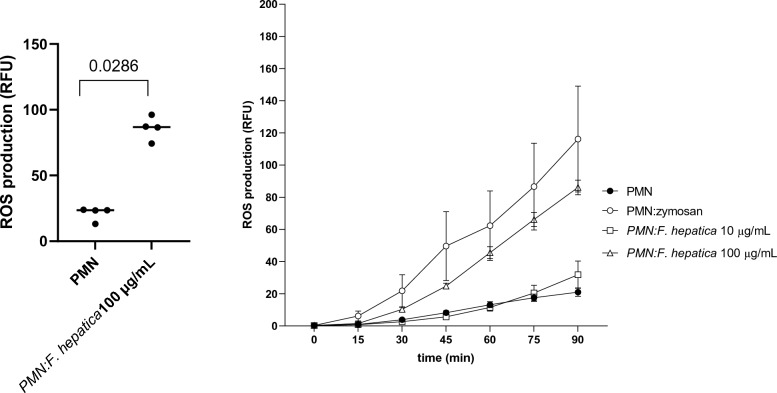
***Fasciola hepatica antigen (FhAg)-stimulated ovine PMN increases intracellular ROS production by oxidation of 2′,7′-dichlorofluorescein diacetate to fluorescent DCF.*** Ovine PMN (*n* = 3) were incubated with *Fh*Ag (10 or 100 μg/mL), in duplicates for 90 min. As controls, plain medium (negative control) and zymosan (1 mg/mL; positive control) were used. **A** Significant differences on ROS production, negative control and *Fh*Ag-stimulated PMN (two tailed t-test *p* = 0.0286). Each dot represents the average/mean of values at 90 min **B** Determination of intracellular ROS production by 90-min temporal analysis was measured via spectrophotometry analysis by using an automated multiplate reader. In both, a clear dose dependent pattern is observed.

*Fh*Ag 100 μg/mL proved to induce an increase of intracellular ROS production as observed at 90 min measurement (*p* = 0.0286; Figure 3A). As positive control, zymosan was used as well-known inducer of PMN respiratory burst activity, while PMN in plain medium were used as negative control. In addition, a 90-min temporal analysis shows dose-dependent intracellular ROS induction observed along different time points, and as expected, zymosan induced increased ROS production from 30 min time point onwards (Figure 3B). In summary, this result show that *F. hepatica* soluble antigen induces intracellular ROS production, commonly associated with NET formation.

### Ovine PMN exposure to *Fh*Ag resulted in neither ECAR-, PER-nor OCR increase

**Figure 4:**
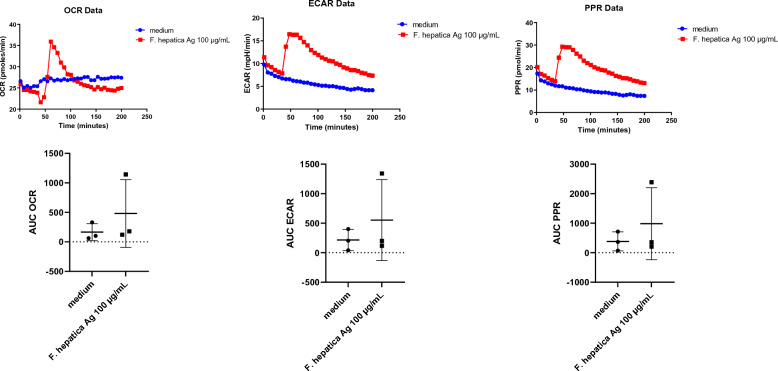
**Ovine PMN activation assay with soluble Fasciola hepatica antigen (FhAg 100 μg/mL).** The mitochondrial respiration profiles (OCR = oxygen consumption rate) and total extracellular acidification rate (ECAR; as defined by the area under the curve; AUC) of control (non-exposed ovine PMN) and ovine PMN stimulated with soluble *Fh*Ag (100 μg/mL). Three biological replicates. Only one animal presented higher OCR, ECAR and proton efflux rate (PER) after stimulation with *Fh*Ag 100 μg/mL, showing that this blood donor was a rather “high responder” while the other animals were rather “low responders”, resulting in a not significant increased response.

Besides, we conducted a series of tests to assess the metabolic activities of *Fh*Ag-exposed ovine PMN (*n* = 3) using an extracellular flux analyser Seahorse XFp® (Agilent, Rathingen, Germany), which assesses OCR associated with mitochondria respiration, ECAR to measure glycolic pathway in vital cells by using cell energy profiles and PER. As illustrated in Figure 4, there was no significant increase in ECAR, OCR and PER values when compared to un-stimulated PMN controls indicating absence of these activities during PMN activation in presence of soluble *Fh*Ag 100 μg/mL. Nonetheless, individual variability was observed since one of the animals showed a much higher response than the other two animals, when PMN were exposed to *Fh*Ag 100 µg/mL picking around 15–20 min after exposure and then decreasing its response to values similar to un-exposed PMN (exemplary curves of OCR, ECAR and PER, Figure 4).

### *Fasciola hepatica* induced leukocyte infiltration and NETs formation in vivo

**Figure 5:**
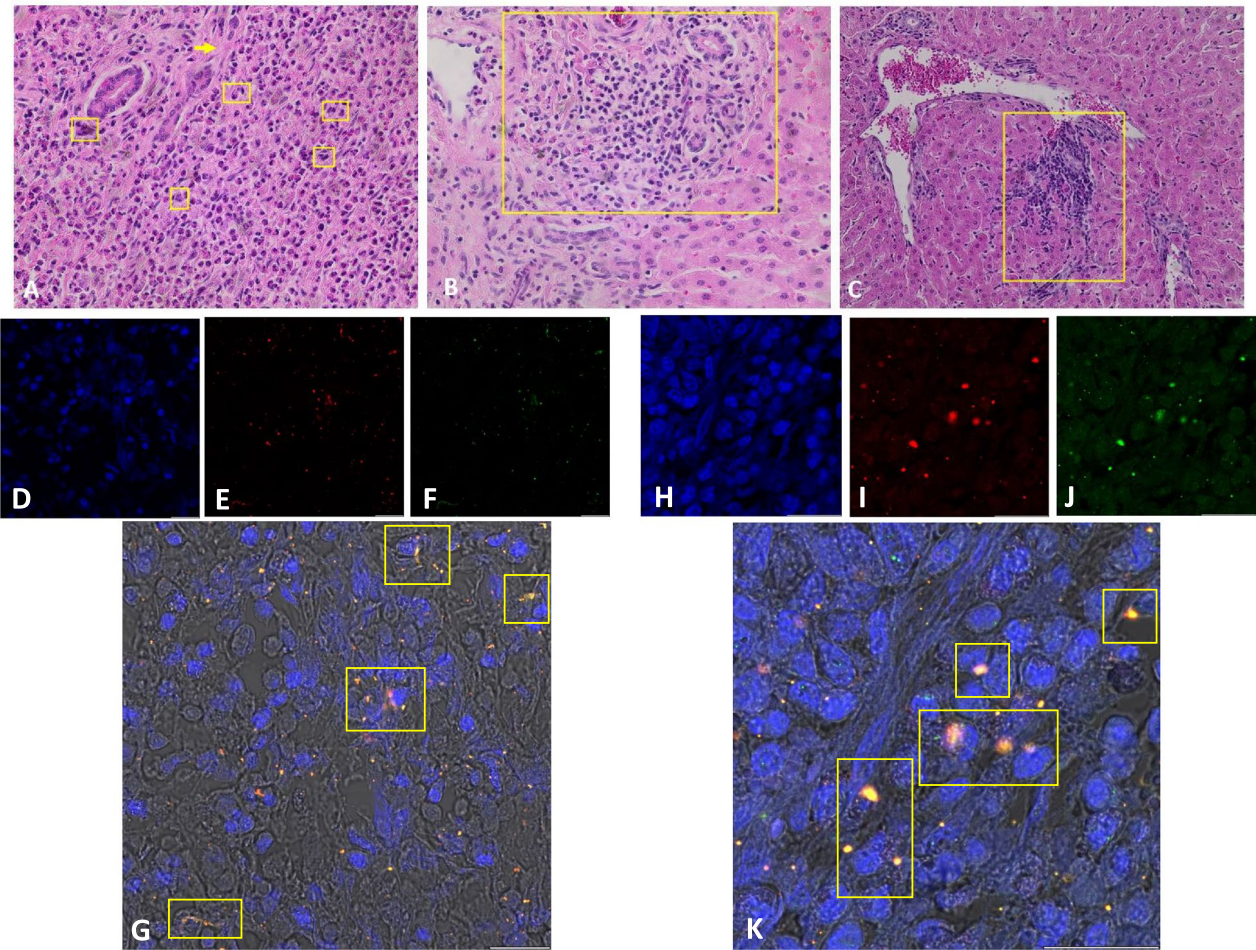
**Histopathological section of ovine liver parasitized with Fasciola hepatica shows different levels of leukocyte infiltration.** **A** multifocal infiltration of inflammatory cells especially eosinophils (yellow boxes) along with fibrotic areas (yellow arrow) in proximity to branch of bile ducts. **B** predominant infiltration of macrophages and PMN in proximity to bile ducts. **C** moderate infiltration of inflammatory cells especially plasma cells and lymphocytes in the portal area. All images: Hematoxylin & Eosin (H&E) staining, ×400 magnification. Immunofluorescence analysis on consecutive sections used for H&E staining. DNA (**D**, **H**; blue), pan-histones (H1, H2A/H2B, H3, H4) (**E**, **I**; red) and neutrophil elastase (NE) (**F**, **J**; green). Depiction of the merge of the three channels (**G** 63X magnification, **K** 40X magnification). 3D imaging video on immunofluorescence analysis of tissue sections provided in Additional file 3.

More importantly, obtained TMA assay data on chemotactic properties of *Fh*Ag in vitro assay was corroborated by histopathological analysis in ovine liver tissue sections of naturally *F. hepatica*-infected animals. Herein, multifocal infiltration of inflammatory leukocytes was observed within hepatic parenchyma, especially eosinophils (Figure 5A) and PMN/macrophages (Figure 5B) were detected alongside with fibrotic and haemorrhagic areas in proximity to branch of bile ducts (Figure 5A and B) as well as infiltration of inflammatory cells especially plasma cells and lymphocytes in the portal area (Figure 5C).

As a next step, we used fluorescence microscopy to visualize formation of ETs in tissue sections, since antibody-based techniques that stain ET-specific markers such as DNA–histone complexes in combination with cell-specific proteins that are frequently found associated with NETs such as NE are needed to confirm ETs release by immunofluorescence microscopy in vivo as stated by de Buhr et al. [38]. As outcome, using this technique, mild-to-moderate positive signals of DNA, histone complex, and NE were observed co-localised within *F. hepatica*-infected liver sections (Figures 5G–K) which is a typical and strong evidence of ET formation. Fine *spr*NETs (Figure 5G) and diffused NETs (*diff*NETs) (Figure 5K) as well as cells in initial phases of NETosis (Figure 5K) were observed in analysed samples, thereby supporting in vitro data. Additionally, 3D imaging of tissue sections support this in vivo data (Additional file 3).

## Discussion

The pathogenesis of fasciolosis is mainly characterized by the active invasion and migration of juvenile *F. hepatica* parasites through the liver parenchyma in order to reach its target settlement area, the bile ducts. During this hepatic migration, *F. hepatica* is permanently exposed to adverse host environments composed by cell barriers, cells of innate and adaptive immune system, complement factors, antibodies, antimicrobial peptides, pro-inflammatory cytokines/chemokines, or other soluble factors [44]. In vivo studies of the early, peritoneal, cellular, and free radical response have already been performed in in rats and mice infected with *Fasciola hepatica* [45, 46]. Nonetheless, investigations on early host innate immune reactions of PMN against *F. hepatica* have scarcely been performed in humans and ruminants [37]. Therefore, we here provide first insights on *F. hepatica*-induced NETosis as early effector mechanism of exposed ovine PMN under different in vitro and in vivo approaches. Overall, we provide evidence on the ability of *F. hepatica*-derived soluble antigen (*Fh*Ag) to trigger ovine NETosis, intracellular ROS production, degranulation, and chemotaxis in vitro, and leukocyte infiltration with concomitant formation of NETs in vivo.

NETs are mainly composed by extracellular decondensed chromatin fibers along with nuclear H1, H2A/H2B, H3, H4, enzymatic granular components, such as NE, MPO, lactoferrin, cathepsin, pentraxin, LL37, and gelatinase among others [21, 24]. In the present study, *F. hepatica*-induced ovine NET formation displayed typical NETs-associated characteristics confirmed by co-localization experiments observing extrusion of extracellular DNA from ovine PMN coated with H1, H2A/H2B, H3, H4 and NE. This data support that PMN incubated with *Fh*Ag soluble antigens receive sufficient stimuli to generate “classical NETs” as shows by previous studies analysing *Fh*Ag soluble antigens-induced NET formation in bovine PMN [37]. These results are also in line with related in vitro studies using parasites soluble antigens evaluating PMN-mediated immune reactions pointing that parasite antigens may lead to a more effective uptake or a better disposability of potential pathogen-associated molecular patterns (PAMP) by PMN [47].

Additionally, SEM analysis confirmed immunofluorescence data showing a clear induction of fine *spr*NETs phenotypes. So far, several phenotypes of NETs have been described induced by parasites, i.e. aggregated NETs (*agg*NETs), *diff*NETs and *spr*NETs [29, 32, 34]. Additional phenotypes including cell free NETs and anchored NETs have been also described to be triggered by parasites [40, 48]. Here, quantification of anchored- and cell free-NETs was performed by PicoGreen®-derived fluorescence analysis observing the release of both, cell free- and anchored NETs in ovine PMN exposed to *Fh*Ag. This is in line with apicomplexan parasites inducing these phenotypes of NETs thereby suggesting either parasite species- or host species-dependent innate immune reactions [18, 49]. However, our results in vitro are consistent with previous studies on *F. hepatica*-induce NETs of bovine PMN showing a rather lower reaction [37] when compared to other trematode parasites inducing NETs such as *F. gigantica* and *S. japonicum* [35, 36].

Additionally, we used live cell three-dimensional holotomographic microscopy to illustrate live cell interactions between ovine PMN after exposure to *Fh*Ag observing as early as 30 min degranulation of exposed PMN. It has been proved that degranulation and release of pro-inflammatory molecules by PMN in vitro and in vivo [50] may cause substantial damage to tissues if not adequately controlled [50, 51], or its release may also be modulated by parasites [52, 53] as in the case of excretory/secretory (ES) molecules of *F. hepatica* which have been demonstrated to actively modulate bovine PMN responses [54]. Then, we determined the chemotactic response of ovine PMN to *Fh*Ag stimulation by using a TMA in vitro resulting in a significant increase of PMN chemotaxis despite the individual variation between PMN donors. Accordingly, data on PMN chemotaxis was corroborated in vivo by histopathological analysis on haematoxylin/eosin-stained ovine livers of naturally *F. hepatica-*infected sheep. Herein, multifocal infiltration of pro-inflammatory cells including eosinophils, PMN, macrophages, plasma cells and lymphocytes was observed within necrotic foci of liver parenchyma in proximity to branch of bile ducts and portal areas confirming the same route of the infiltrating inflammatory cells as described elsewhere [44]. Then, we used fluorescence microscopy to visualize and confirm the formation of ETs by using antibody-based techniques that stain ET-specific markers such as DNA–histone complexes in combination with cell-specific proteins that are frequently found associated with NETs such as NE [38]. As outcome, mild-to-moderate positive signals of DNA, histone complex, and NE were observed co-localised within *F. hepatica*-parasitized liver tissue which is a typical and strong evidence of ET formation. Fine *spr*NETs phenotypes we here observed in vivo along with *diff*NETs thereby corroborating in vitro data.

More recently, a study on *Besnoitia besnoiti*-induced NETosis demonstrated that histone H2A and NETs induced endothelial dysfunction, cell death and endothelium damage in vitro [55] thereby proving also adverse NETosis-derived effects on blood vessels. Thus, we hypothesize that NET-formation by PMN infiltrating the hepatic parenchyma and bile ducts in response to *F. hepatica* might have a role in the pathogenesis of acute and chronic fasciolosis, most probably during migrating phase of the immature juveniles throughout liver parenchyma, when major cellular inflammatory infiltration occurs.

Irrespective of NETosis-derived adverse effects on endothelium or other tissues [55–57], it is important to consider NETs-derived anti-parasitic mechanisms against metazoan parasites as demonstrated previously against trematodes [35, 36]; and nematodes [28, 29, 31, 32, 34]. Consequently, NETosis against either protozoan or metazoan parasites is crucial for adequate host innate defence mechanisms during the course of many parasitic diseases and might also be pivotal in ruminant fasciolosis [37].

Finally, a dose dependent induction of intracellular ROS production in ovine PMN exposed to *Fh*Ag was here observed as soon as 30 min post stimulation onwards until 90 min measurement. An increase of intracellular ROS have also been described in the bovine system under same stimuli but in a rather lower intensity [37], thereby suggesting different host-specific responses to *Fh*Ag. Meanwhile, in ovine, increase of hydrogen peroxide (H_2_O_2_) and nitric oxide (NO) levels have been reported on peritoneal granulocytes of *F. hepatica*-infected sheep in early stages of the infection [58]. Furthermore, in vitro studies performed in Indonesian thin-tail (ITT) sheep showed an antibody-dependent superoxide radical cytotoxicity against juvenile *Fasciola gigantica* but not juvenile *Fasciola hepatica* [59] pointing different immune responses according to host breed. However, mitochondrial-derived OCR, ECAR and PER metabolic activities of ovine PMN did not show significant increases, therefore suggesting low grade of such metabolic activities during ovine PMN activation upon exposure to *Fh*Ag. Nonetheless, different metabolic routes, including glycolysis with ATP generation, are required to fulfil energetic, biosynthesis, and defence functions of mammalian PMN [60–62]. Recently, it has been demonstrated that mammalian PMN can additionally rely on TCA cycle, oxidative phosphorylation (OXPHOS), pentose phosphate pathway (PPP), and fatty acid oxidation (FAO) for energy [63, 64]. Overall, here we present new insights into early host innate immune response in vivo and in vitro of ovine PMN driven by *F. hepatica* considering ovine hosts as relevant and highly susceptible animals to fasciolosis. Ex vivo evidence on the role of NETs triggered by parasites antigens on humans with *Opisthorchis viverrine*-derived hepatobiliary abnormalities have been recently published [65]. In this line, we demonstrate for the first time the ability of soluble *Fh*Ag to trigger ovine NETs, chemotaxis, and intracellular ROS production in vitro as well as leukocyte infiltration with NET formation in vivo. Therefore, ovine NETs might not only facilitate parasite killing by other recruited leukocytes within affected tissues but also contributing to the pathogenesis of fasciolosis in vivo through NETs-derived tissue damage.

### Supplementary Information

**Additional file 1. Spectrofluorometric analysis performed by PicoGreen®-derived fluorescence intensities for anchored and cell free NETs of PMN exposed to FhAg (10 and 100 μg/mL) for 120 min.**

**Additional file 2. Live cell 3D holotomographic analysis show images of degranulation of ovine PMN as early as 30 min after stimulation evolving continuously thereafter.** From this time point, ovine PMN granules are disperse in the cytoplasm but from 4 h after exposure higher refractive index (RI) signals are observed closer to the nucleus (red rectangle). At 12 h some cells are disintegrated while others remained with compacted nucleus.

**Additional file 3. 3D imaging on in vivo NETs.** 3D imaging immunofluorescence analysis of ovine liver tissue sections from naturally *Fasciola hepatica-*infected animals show co-localization of DNA (blue), global histones (H1, H2A/H2B, H3, H4) (red) and neutrophil elastase (NE; green) originating from leukocytes which infiltrated the liver parenchyma. 63X magnification.

## Data Availability

The datasets used during the current study are available from the corresponding author on reasonable request.
