# Correction: *Fasciola hepatica* soluble antigens (*FhAg*) induce ovine PMN innate immune reactions and NET formation in vitro and in vivo

**DOI:** 10.1186/s13567-024-01297-8

**Published:** 2024-03-26

**Authors:** Tamara Muñoz-Caro, Marcela Gómez-Ceruti, Liliana M. R. Silva, Daniel Gutiérrez-Expósito, Henrik Wagner, Anja Taubert, Carlos Hermosilla

**Affiliations:** 1 Escuela de Medicina Veterinaria, Facultad de Medicina Veterinaria y Recursos Naturales, Universidad Santo Tomás, Talca, Chile; 2 Centro de Investigación de Ovinos Para El Secano OVISNOVA, Facultad de Medicina Veterinaria y Recursos Naturales, Universidad Santo Tomás, Talca, Chile; 3 Centro de Investigação Interdisciplinar Egas Moniz (CiiEM), Instituto Universitário Egas Moniz, Caparica, Portugal; 4 MED-Mediterranean Institute for Agriculture, Environment and Development & CHANGE-Global Change and Sustainability Institute, Universidade de Évora, Evora, Portugal; 5 Departamento de Sanidad Animal, Instituto de Ganadería de Montaña (CSIC-ULE), Facultad de Veterinaria, Universidad de León, Campus de Vegazana S/N, 24071 León, Spain; 6 Veterinary Clinic for Reproduction and Neonatology, Justus Liebig University Giessen, Giessen, Germany; 7 Institute of Parasitology, Faculty of Veterinary Medicine, Justus Liebig University Giessen, Giessen, Germany

**Correction: Veterinary Research (2023) 54:105** 10.1186/s13567-023-01236-z

Following publication of the original article [1], we have added the author Carlos Hermosilla to Correspondence.

The original article has been corrected.
