# Supplementary figures and images for: Fasciola hepatica soluble antigens (FhAg) induce ovine PMN innate immune reactions and NET formation in vitro and in vivo
Source: Vet Res. 2023 Nov 12;54:105. doi: 10.1186/s13567-023-01236-z (PMC10642000; doi:10.1186/s13567-023-01236-z)

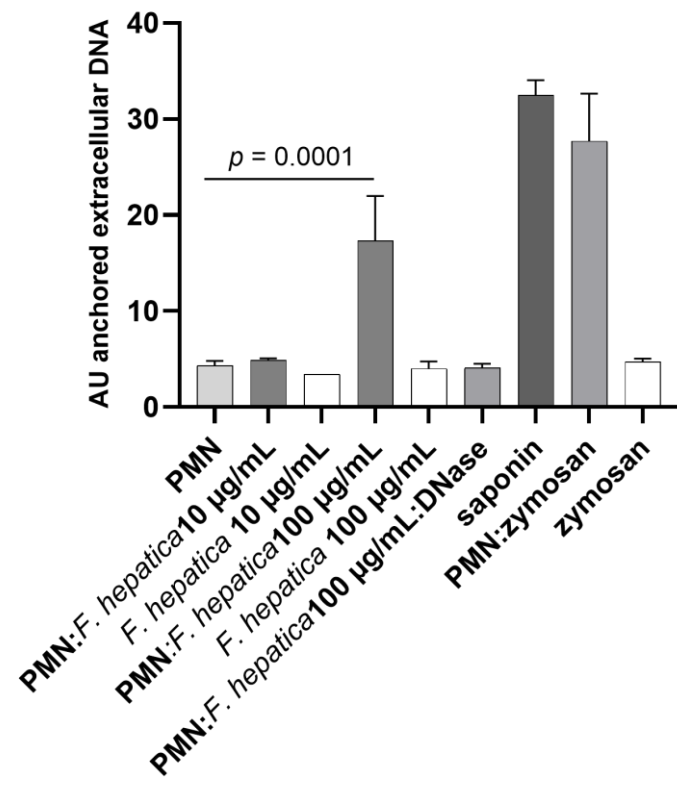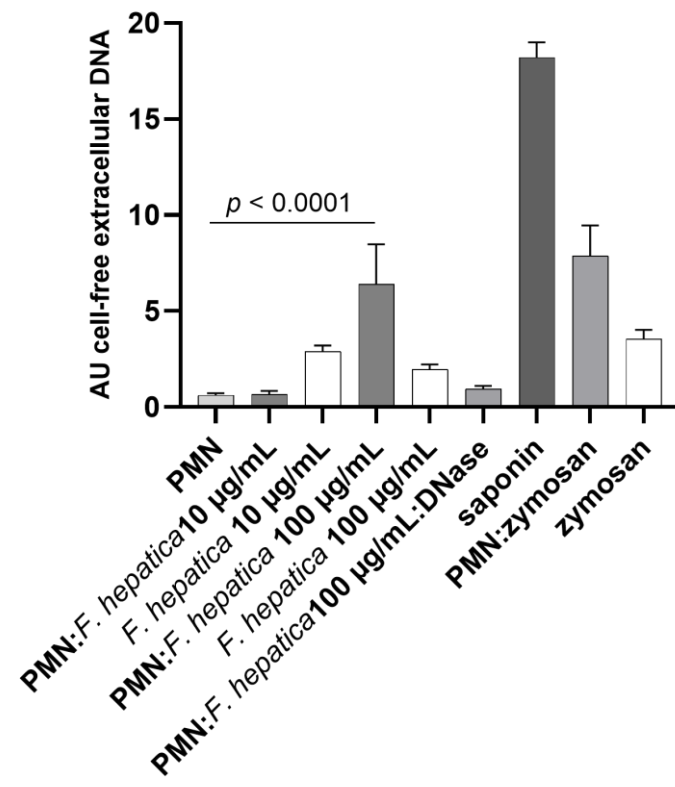

Supplement: Supplementary file 1 — Additional file 1. Spectrofluorometric analysis performed by PicoGreen®-derived fluorescence intensities for anchored and cell free NETs of PMN exposed to FhAg (10 and 100 μg/mL) for 120 min. [file 13567_2023_1236_MOESM1_ESM.pdf]

3D Rendering  
Digital Staining

0 min

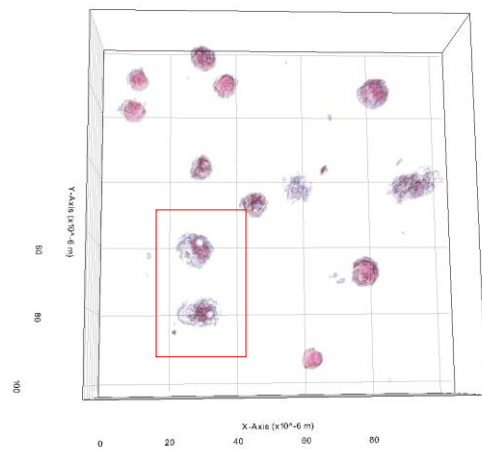

5 min

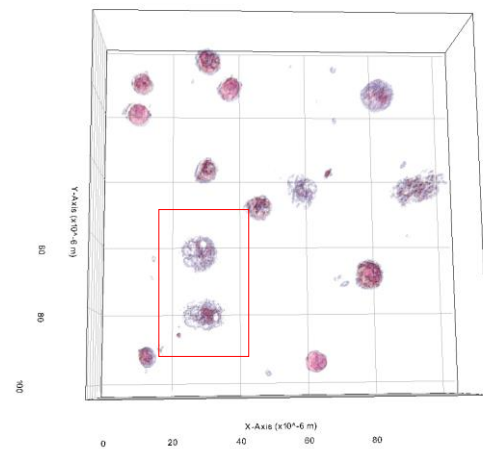

30 min

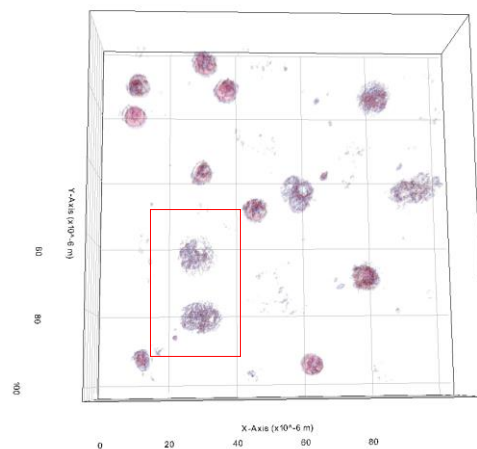

60 min

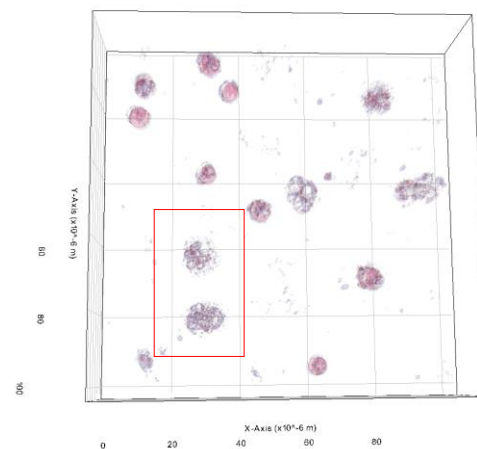

2 h

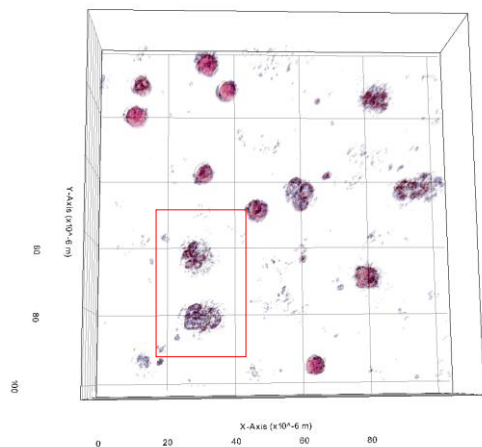

4 h

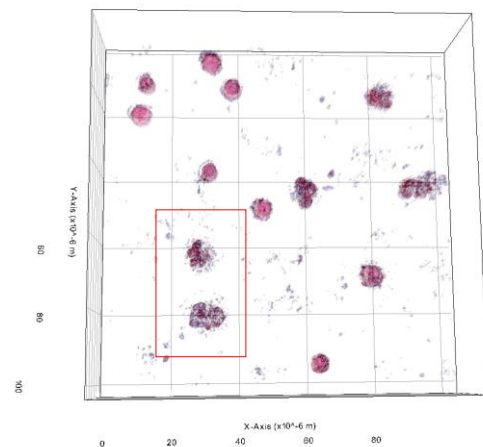

8 h

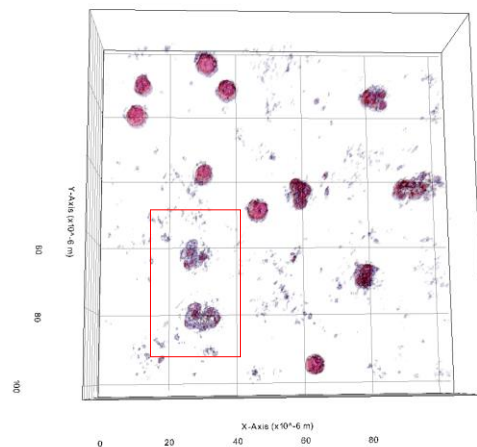

12 h

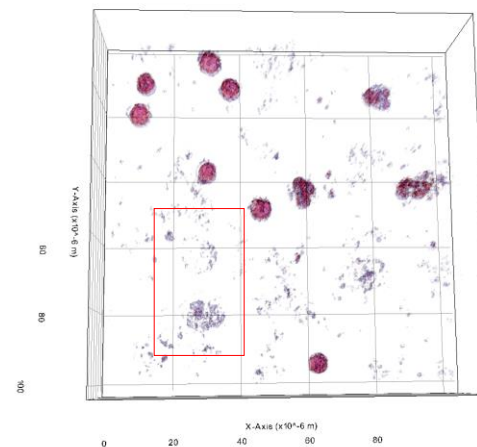

3D Rendering  
Digital Staining

Supplement: Supplementary file 2 — Additional file 2. Live cell 3D holotomographic analysis show images of degranulation of ovine PMN as early as 30 min after stimulation evolving continuously thereafter. From this time point, ovine PMN granules are disperse in the cytoplasm but from 4 h after exposure higher refractive index (RI) signals are observed closer to the nucleus (red rectangle). At 12 h some cells are disintegrated while others remained with compacted nucleus. [file 13567_2023_1236_MOESM2_ESM.pdf]

## Slide 1
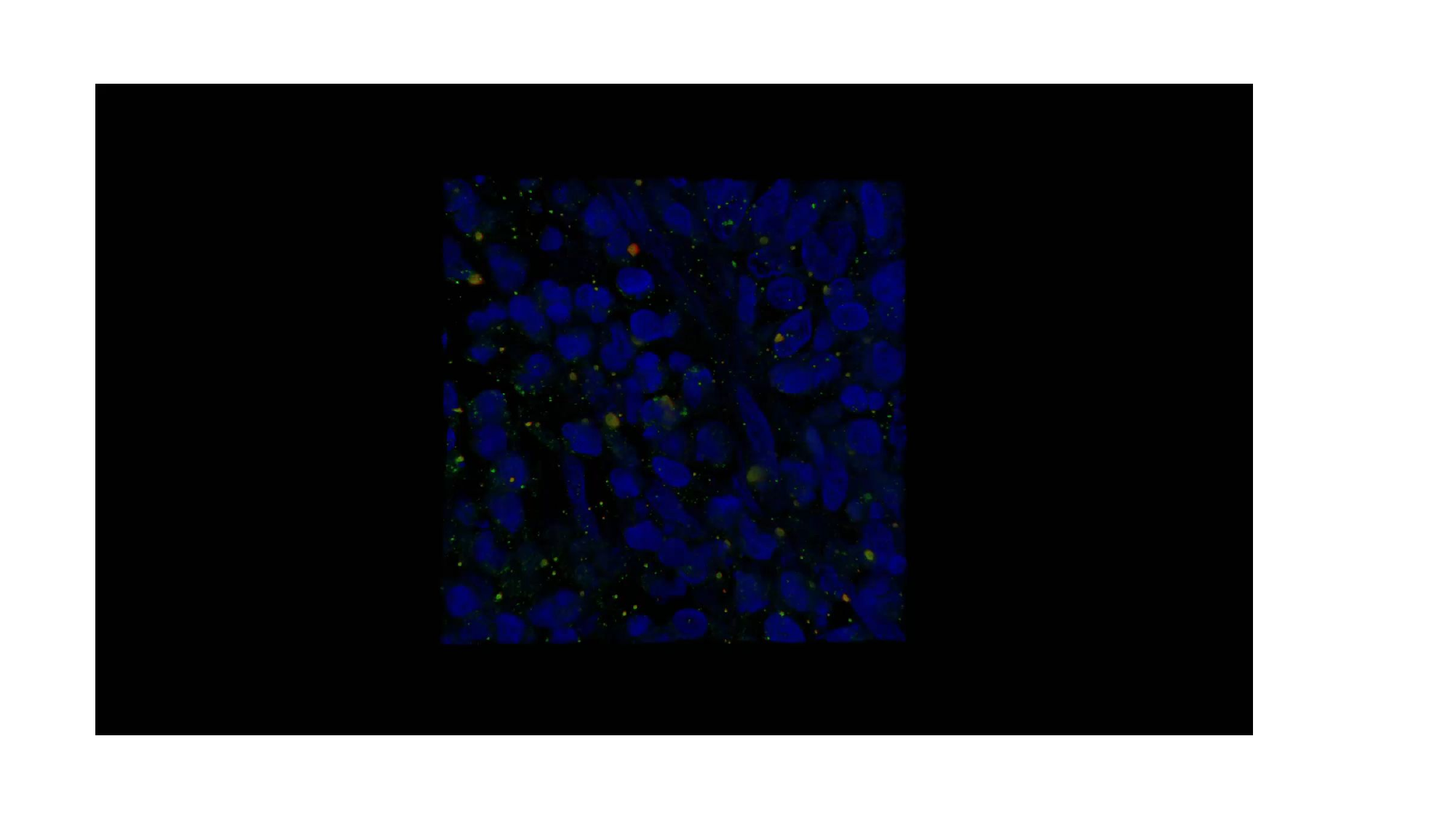

Supplement: Supplementary file 3 — Additional file 3. 3D imaging on in vivo NETs. 3D imaging immunofluorescence analysis of ovine liver tissue sections from naturally Fasciola hepatica-infected animals show co-localization of DNA (blue), global histones (H1, H2A/H2B, H3, H4) (red) and neutrophil elastase (NE; green) originating from leukocytes which infiltrated the liver parenchyma. 63X magnification. [file 13567_2023_1236_MOESM3_ESM.pptx]
